# Testing Hypoxia in Pig Meniscal Culture: Biological Role of the Vascular-Related Factors in the Differentiation and Viability of Neonatal Meniscus

**DOI:** 10.3390/ijms222212465

**Published:** 2021-11-18

**Authors:** Barbara Canciani, Valentina Rafaela Herrera Millar, Margherita Pallaoro, Lucia Aidos, Federica Cirillo, Luigi Anastasia, Giuseppe Maria Peretti, Silvia Clotilde Modina, Laura Mangiavini, Alessia Di Giancamillo

**Affiliations:** 1 IRCCS Istituto Ortopedico Galeazzi, 20161 Milan, Italy; canciani.barbara@hsr.it (B.C.); giuseppe.peretti@unimi.it (G.M.P.); 2 Department of Veterinary Medicine, University of Milan, 26900 Lodi, Italy; valentina.herrera@unimi.it (V.R.H.M.); margherita.pallaoro@studenti.unimi.it (M.P.); lucia.aidos@unimi.it (L.A.); silvia.modina@unimi.it (S.C.M.); alessia.digiancamillo@unimi.it (A.D.G.); 3 Stem Cells for Tissue Engineering Laboratory, IRCCS Policlinico San Donato, 20097 San Donato Milanese, Italy; federica.cirillo@grupposandonato.it (F.C.); anastasia.luigi@unisr.it (L.A.); 4 Faculty of Medicine and Surgery, Università Vita-Salute San Raffaele, 20132 Milan, Italy; 5 Department of Biomedical Sciences for Health, University of Milan, 20133 Milan, Italy

**Keywords:** hypoxia, meniscus, pig, fibro-chondrocytes, differentiation, HIF-1α

## Abstract

Menisci play an essential role in shock absorption, joint stability, load resistance and its transmission thanks to their conformation. Adult menisci can be divided in three zones based on the vascularization: an avascular inner zone with no blood supply, a fully vascularized outer zone, and an intermediate zone. This organization, in addition to the incomplete knowledge about meniscal biology, composition, and gene expression, makes meniscal regeneration still one of the major challenges both in orthopedics and in tissue engineering. To overcome this issue, we aimed to investigate the role of hypoxia in the differentiation of the three anatomical areas of newborn piglet menisci (anterior horn (A), central body (C), and posterior horn (P)) and its effects on vascular factors. After sample collection, menisci were divided in A, C, P, and they were cultured in vitro under hypoxic (1% O_2_) and normoxic (21% O_2_) conditions at four different experimental time points (T0 = day of explant; T7 = day 7; T10 = day 10; T14 = day 14); samples were then evaluated through immune, histological, and molecular analyses, cell morpho-functional characteristics; with particular focus on matrix composition and expression of vascular factors. It was observed that hypoxia retained the initial phenotype of cells and induced extracellular matrix production resembling a mature tissue. Hypoxia also modulated the expression of angiogenic factors, especially in the early phase of the study. Thus, we observed that hypoxia contributes to the fibro-chondrogenic differentiation with the involvement of angiogenic factors, especially in the posterior horn, which corresponds to the predominant weight-bearing portion.

## 1. Introduction

Menisci are semi-circular, wedge-like structures located in the medial and the lateral side of the knee joint and play a fundamental role in knee stability, shock absorption, and load distribution. Menisci largely consist of fibrocartilage tissue, where cells are capable of synthesizing a variety of matrix molecules, such as collagens, proteoglycans, and non-collagenous proteins [1,2,3,4]. The meniscus is divided into an inner and an outer region [5]. The inner portion (known also as white-white zone) is avascular, tapering to a thin free edge, and it is close to the condyles; whereas, the outer part (known also as red-red zone) receives blood supply, and it represents the thickest border close to the synovial joint capsule [1,2,3,4,6]. The two portions of the meniscus differ in the cellular composition and in the morphology of the extracellular matrix. The outer region is composed of fibroblast-like cells, which produce a matrix rich in collagen type I and poor in glycosaminoglycans (GAGs) [7,8]. Conversely, the inner portion resembles the hyaline cartilage, and it contains chondrocyte-like cells and a matrix abundant in type II collagen, in combination with small amounts of type I collagen and GAGs [9,10]. The meniscus can be anatomically and functionally subdivided in three portions: the anterior horn (A), the central body (C), and the posterior horn (P) [11,12]. Collagen type II and GAGs are particularly abundant in the anterior horn to resist to compression during loading. Collagen type I is more expressed in the posterior horn, increasing resistance to tensile forces during locomotion [2,3]. Healing potential of the meniscus is limited due to the low blood supply especially in adults [13,14,15,16]. Generally, lesions of the outer part can repair [17,18,19,20]; whereas, the healing is impaired in the avascular inner zone [21,22,23,24,25]. Newborn menisci are fully vascularized, and blood supply progressively decreases within the first three months of life [2,4,13,26]. Around 10 years of age, the vascularization occurs in 10–30% of the meniscus; while, in adults blood supply is limited at 10–25% of the peripheral region [2,4,26]. Invasion and maintenance of new blood vessels is controlled by a balanced variety of angiogenic and anti-angiogenic factors [27,28]. In young pig menisci, the switch from fibroblast-like to the fibrochondrocyte phenotype is characterized by a high expression of the anti-angiogenic factor endostatin, which is associated with vascular reduction [29]. At the same time, vascular endothelial growth factor (VEGF), an important pro-angiogenic factor, is expressed in meniscal endothelial cells and in fibrochondrocytes [30].

Hypoxia is one of the features of the meniscal environment that influences development and maturation of the fibrocartilage phenotype of the avascular inner zone. Cells survive and produce extracellular matrix in a hypoxic environment, as shown in different mouse models [31,32,33,34]. Several experiments have been focused on chondrogenesis (i.e., chondrocyte growth and survival) and matrix formation, either in pellet or scaffold cultures, under hypoxia conditions [31,33,35,36]. These studies demonstrated that hypoxia, and in particular hypoxia-inducible factors, play a crucial role in chondrocytes’ survival and differentiation [31,33,37].

Our recent study revealed that hypoxia worked as a booster to achieve meniscal cell maturation on the whole meniscal tissue [38]. The aim of this novel study is to assess if hypoxia differently regulates meniscal cell proliferation and differentiation based on the anatomical position. Thus, we cultured in vitro the three different portions of neonatal pig menisci (anterior horn, central body, and posterior horn) in normoxia (21% O_2_) or hypoxia (1% O_2_) conditions for up to 14 days. In our study we used neonatal cells, which were committed to assume a fibro-chondrocyte-like phenotype, but they were still immature. At the end of the experimental time points, we analyzed samples by morphology and gene expression, with particular focus on markers of meniscal extracellular matrix and vascular-related factors.

## 2. Results

### 2.1. Morphological Analyses

**Figure 1:**
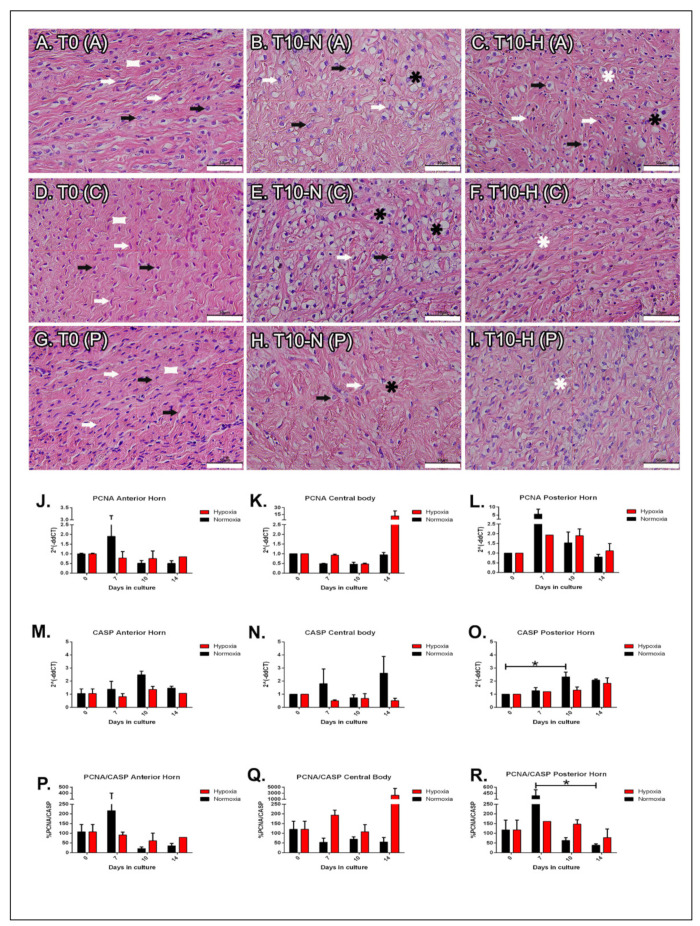
Meniscus morpho-functional characterization during early development. T0 (**A**,**D**,**G**), T10 normo- (**B**,**E**,**H**), and T10 hypoxic (**C**,**F**,**I**) HE staining showing meniscal morphological features and cells distribution in anterior (**A**–**C**), central (**D**–**F**), and posterior (**G**–**I**) portions. White arrows emphasize darker and elongated nuclei, black arrows highlight lighter and irregular-round shaped nuclei. Black and white asterisks indicate large and small lacunae, respectively. White squares point to acidophilic matrix. Bars: 50 μm. J-R: time course of qPCR for PCNA (**J**–**L**), CASP3 (**M**–**O**), and PCNA/CASP3 ratio (**P**–**R**) mRNA expression in normo- (black histogram) and hypoxic (red histogram) conditions in the three portions of the meniscus. Data are expressed as mean ± s.e.m. * = *p* value < 0.05.

**Figure 2:**
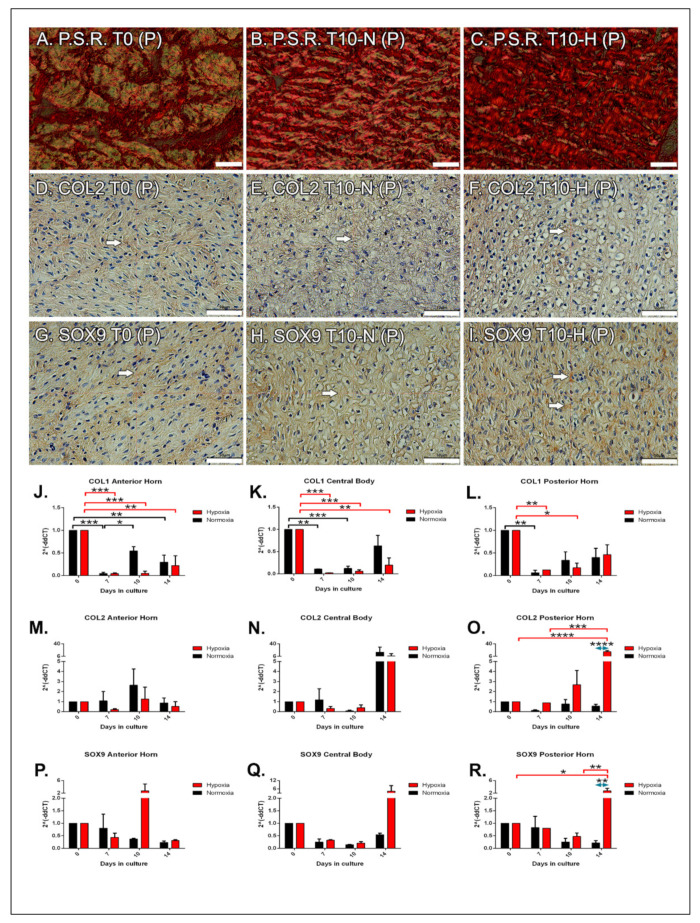
Extracellular matrix composition. Collagen type I location by picrosirius staining (P.S.R.) at T0 (**A**), T10 normoxic (**B**), and hypoxic (**C**) conditions. Immunohistochemistry for collagen type II expression at T0 (**D**), T10 normoxic (**E**), and hypoxic (**F**) conditions. SOX9 expression at T0 (**G**), T10 normoxic (**H**), and hypoxic (**I**) conditions. All microphotographs were taken in the posterior horn. White arrows highlight the positive stain in the matrix or nuclei. Bars: 50 μm. Time course of qPCR for COL1A1 (**J**–**L**), COL2A1 (**M**–**O**), and SOX9 (**P**–**R**) mRNA expression in normo- (black histogram) and hypoxic (red histogram) conditions in the three portions of the meniscus. Data are expressed as mean ± s.e.m. * = *p* value < 0.05; ** = *p* value < 0.01; *** = *p* value < 0.001; **** = *p* value < 0.0001. Blue left right double arrow indicates significant difference between hypoxia and normoxia at the given experimental time.

Morphological analyses are reported in Figure 1. At T0 newborn meniscus (T0) was rich in cells, especially in the inner zone, that were homogenously distributed in the tissue (Figure 1A,D,G, arrows). The meniscal cell phenotype appeared heterogeneous as some fibroblast-like cells had dark, slender nuclei (Figure 1A,D,G, white arrows); while others had larger, less dark, and uneven round shaped nuclei (Figure 1A,D,G, black arrows). Both cell types were immersed in an acidophilic fibrous matrix (Figure 2A,D,G, white squares). The seventh day of culture represented an intermediate stage, with no relevant morphological changes, except for an evident cellular reduction in the posterior horn in normoxia. Cell loss was also noted at T10, particularly marked in normoxia (Figure 1B,E,H); the nuclei were still heterogeneous (Figure 1B,E,H, white arrows for fibroblast-like cells and black arrows for chondrocyte-like cells), and lacunae enlarged as they progressed to the inner zone (Figure 1B,E,H, black asterisks), losing the cellular organization observable in previous experimental time points. Conversely, in A, C, P, at 10 days of hypoxic culture, cells continued to be immersed in lacunae of small dimensions (Figure 1C,F,I, white asterisks). In the longest experimental time point, the tissue showed lacunae of variable dimensions, and many of them appeared empty both in normoxia and hypoxia (images not presented in the panel).

### 2.2. Molecular Analysis of Cell Viability

Cell proliferative capacity was assessed by evaluating the expression of the PCNA (Figure 1J–L). At T7 in normoxia, PCNA expression increased in the two horns, then it quickly decreased at T10 (not significant) (Figure 1J,L). In the central body in normoxic conditions, PCNA expression remained constant over time, but it tended to rise at T14 under hypoxia (not significant) (Figure 1K). The apoptosis index was studied by CASP expression (Figure 1M–O). In the anterior horn, CASP3 expression is lower in hypoxia at each experimental time point compared to normoxia (Figure 1M). In the central body, the CASP3 gene remained constantly lower in hypoxia compared to T0, while in normoxia it had a fluctuating (not significant) trend (Figure 1M–N). On the other hand, the posterior horn shows a significant increase in CASP3 expression at T10 under normoxic conditions (*p* < 0.05) (Figure 1O). The Proliferation/Apoptosis Ratio (PAR) was assessed by calculating the ratio between PCNA and CASP3 [39]. In the anterior horn, the trend remained constant over time under hypoxic conditions, while in normoxia there was a peak after seven days (not significant) which rapidly decreased after 10 days (Figure 1P). In the central body the trend remained constant up to T10 in both experimental conditions, but an increase in vitality was observed in hypoxia at T14 (Figure 1Q). The posterior horn showed a trend comparable to the anterior horn; however, from 7 to 14 days a significant decrease occurred in normoxia (*p* < 0.05) (Figure 1R).

### 2.3. Histological Analysis: Type I Collagen

We decided to show only the posterior horn because this portion of the medial meniscus is the most involved in the shock absorbing capacity (Figure 2). Picrosirius Red stain was used to analyze type I collagen fibers (red color, Figure 2A–C). Weak positivity was observed at T0 in the three portions A, C, P (Figure 2A). From T7 up to T10 there was an increase in positivity under both experimental conditions, and in all the three portions: in particular, the posterior horn was characterized by a visible increase in hypoxia compared to normoxia (Figure 2B,C). At the last experimental time, the production of the protein in the three portions decreased, with no substantial differences observed between treatments and the previous time points.

### 2.4. Immunohistochemical Analysis: Type II Collagen

The distribution of type II collagen fibers was examined by immunohistochemistry (brown color). At T0 the matrix was characterized by the presence of the protein (Figure 2D, white arrow). After 7 days in culture, the positive signal started decreasing in both conditions in the three parts, then it became slightly more pronounced at T10 (Figure 2E,F, white arrow), and ultimately it decreased again at T14. No evident qualitative differences were found between the two treatments.

### 2.5. Immunohistochemical Analysis: SOX9

SOX9 expression was analyzed to assess the early phases of chondrogenesis (positive immunostaining in brown color). The three portions showed a slight positivity for anti-SOX9 antibody at T0 (Figure 2G, white arrow). At T7 no detectable changes were observed either between the two treatments, nor compared to T0. At T10, SOX9 expression increased under hypoxic conditions compared both to T0 and to the normoxic time point T10 (Figure 2H,I, white arrows); however, at T14 an opposite trend occurred in both experimental groups. The central body in both the two experimental groups had a similar trend with the positivity remaining almost unchanged up to T10, and it increased to T14.

### 2.6. Molecular Analysis

COL1A1 expression decreases in the three portions compared to T0 (Figure 2J–L). In the anterior horn, the decrease was significant already after 7 days in both hypoxia and normoxia (*p* < 0.001); in hypoxia, this reduction remained constant up to 14 days (no significant differences vs. T7), while in normoxia at T10 there was a significant increase in COL1A1 compared to T7, which remained constant at T14 (no significant differences vs. T10) (Figure 2J). Concerning the central body, the reduction of COL1A1 remained significant up to T14 only in the hypoxic samples (*p* < 0.01), while in normoxia at T14 COL1A1 expression increased without reaching a significant difference compared to T0 (Figure 2K). In the posterior horn, the reduction of COL1A1 in hypoxia remained significant up to T10 (*p* < 0.05); whereas, in normoxia this decrease persisted only up to T10 (Figure 2L). At the longest time point, COL1A1 expression started rising.

COL2A1 expression in the anterior horn did not show significant changes in the 14 experimental days, but had a peak at T10 in both experimental conditions, though without reaching a significant difference (Figure 2M). In the central body, COL2A1 expression did not significantly change up to T10, neither considering the time nor the treatment; however, T14 was characterized by a rise in COL2A1 expression in both sets of samples (Figure 2N). The posterior horn mimicked the other two portions up to T10; however, COL2A1 expression significantly increased at T14 under hypoxia (*p* < 0.001) (Figure 2O).

SOX9 expression remained constant in the anterior horn and in the central body over time in normoxia, while hypoxia determines a rise in gene expression at T10 and T14, respectively (not significant) (Figure 2P–Q). SOX9 expression in the posterior horn was characterized by a significant increase at T14 compared both to T0 (*p* < 0.05) and T10 hypoxic samples (*p* < 0.01), and to T14 normoxic condition (*p* < 0.01) (Figure 2R).

### 2.7. Immunohistochemical Analysis: Endostatin

**Figure 3:**
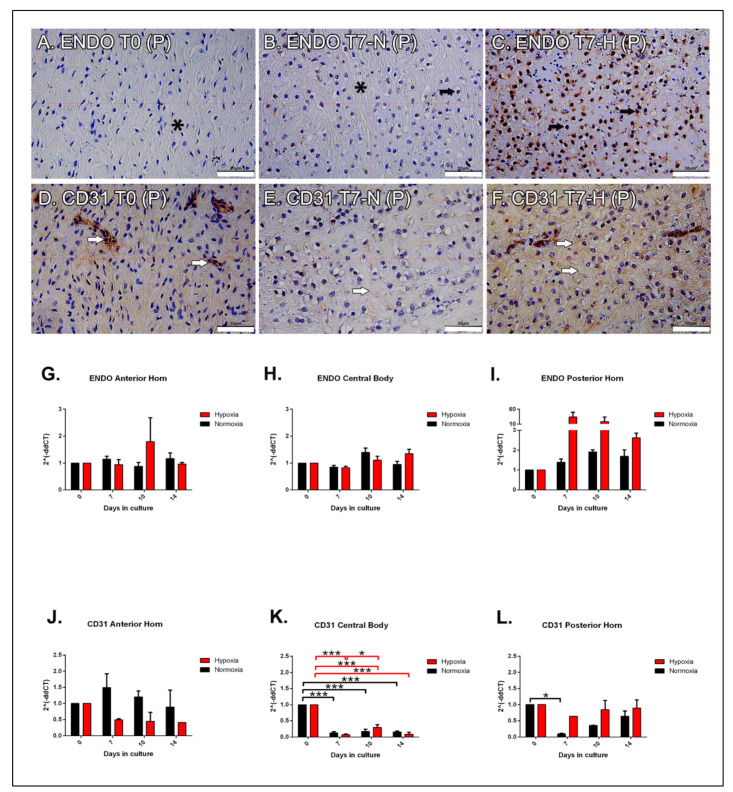
Expression of vascular-related factors. Immunohistochemistry for endostatin expression at T0 (**A**), T7 normoxic (**B**), and hypoxic (**C**) conditions. Black arrows and asterisk in (**A**,**B**) highlight the negative staining in the nuclei and in the matrix, respectively. Black arrows in B and C point at the positive nuclear stain. Immunohistochemistry for CD31 expression at T0 (**D**), T7 normoxic (**E**), and hypoxic (**F**) conditions. White arrows point at the positive blood vessels (**D**), and negative (**E**) or positive (**F**) matrix stain. All microphotographs were taken in the posterior horn. Bars: 50 μm. Time course of qPCR for ENDO (**G**–**I**) and CD31 (**J**–**L**) mRNA expression in normoxic (black histogram) and hypoxic (red histogram) conditions in the three portions of the meniscus. Data are expressed as mean ± s.e.m. * = *p* value < 0.05; *** = *p* value < 0.001.

At T0, endothelial cells appeared scarcely marked at nuclear level, and the signal in the extracellular matrix was almost negative in all portions (Figure 3A, black asterisk). At T7, the staining was similar to T0 in the anterior horn and in the central body, while an increased expression was observed in the posterior horn, where the normoxic sections appeared weakly positive in the nuclei of the inner zone (Figure 3B, black arrow and asterisk for nuclei and negative matrix, respectively). In hypoxia, cell nuclei stained positive in all the samples, particularly in the inner zones and in the matrix (Figure 3C, black arrows). At T10 and T14 the signal progressively reduced, especially in normoxic conditions.

### 2.8. Immunohistochemical Analysis: CD31

In all samples, the endothelial cells stained positive for CD31. At T0 the signal was homogeneously distributed throughout the tissue, consistent with the diffuse vascularization of the meniscus at this age (Figure 3D, white arrows). From T7 up to T14, CD31 protein expression was reduced in all the samples when compared to T0. In hypoxic samples at T7, CD31 positive vessels were observed throughout the three portions (A, C, P) of the tissue. In particular, in the hypoxic posterior horn (Figure 3F, white arrows) a strong matrix immunostaining was detected when compared to normoxia (Figure 3E white arrow). At T10 a weaker CD31-positive staining was observed both in normoxic and hypoxic conditions in A, C, and P; similarly to the T14 condition where the staining was further reduced and almost negative (data not shown).

### 2.9. Molecular Analysis

In the anterior horn, endostatin expression did not significantly differ between the two experimental conditions; however, an increasing trend of its expression was noted at T10 (Figure 3G). In the central body, the temporal variable had an impact on the endostatin levels; hypoxic samples showed a slight decrease in gene expression at T10, followed by a constant increase. On the other hand, endostatin levels in normoxia increased up to T10, but they decreased at T14, though without a significant difference (Figure 3H). The posterior horn showed a different trend, as the expression of endostatin remained constant in normoxia, while it reached a peak in hypoxia at T7, followed by a marked decreased at the longer time points (Figure 3I). Conversely, CD31 levels have a different trend in the three portions (Figure 3J–L). In the anterior horn, CD31 expression was markedly reduced in hypoxia, while it tended to rise in normoxia at T7 (not significant) (Figure 3J). In the central body, CD31 levels significantly reduced at T7, and they remained steady in the other two experimental times in both conditions (*p* < 0.001) (Figure 3K). In the posterior horn, CD31 expression remained constant over the time without significant changes; whereas, its levels significantly decreased in normoxic samples at T7 (*p* < 0.05) (Figure 3L).

## 3. Discussion

This study focused on in vitro cultures of meniscal tissue from newborn piglets to evaluate how hypoxia may modulate the differentiation of the three portions (A, C, P) of meniscus, both in terms of morphology and production of typical tissue matrix, as well as in terms of vascularization.

One of the major problems of the repair strategies in the inner avascular and fibro-cartilaginous zone of the meniscus is related to the control of the cellular phenotype. Fibro-chondrocytes are crucial for the biomechanics and integrity of the meniscus itself, as they are responsible for the secretion of a specific matrix. Many pathological phenomena lead to the dedifferentiation of fibro-chondrocytes, that is also frequently observed in tissue culture, making the regeneration strategies more difficult. More in general, it has long been known that a big issue about in vitro culture is that serial subculture of chondrocytes and/or fibro-chondrocytes leads to loss of phenotype [40,41].

The meniscus physiologically survives in a hypoxic environment; thus, fibro-chondrocytes are cells that have adapted to the low-oxygen tension, and supporting this idea recent evidence demonstrated that hypoxia promotes the human and bovine chondrocyte phenotype differentiation at 5% oxygen tension [42,43].

Thus, hypoxia can be used as a stimulus to overcome the problem related to the de-differentiation in human meniscal cells [44], and it can be applied to promote the maturation of neonatal piglet meniscal cells [38]. Furthermore, it has been observed that the change in cell phenotype coincides with a change in the vascularity of the tissue in the meniscus [29,45]; therefore, in this study we focused our attention on two particular vascular factors, the pro-angiogenic factor CD31 and the anti-angiogenic factor endostatin. The choice to divide the meniscus into three portions was due to the slightly different and fully recognized micro- and macroscopic characteristics observed among the three zones [46]. In a recent study of our group, we found that hypoxia acted as boost for meniscal differentiation; however, the study was performed on the whole meniscus, and no regional differentiation has been evaluated [38]. The data presented in this manuscript showed that as concerns cell viability, hypoxia tended to retain the initial phenotype of cells, although some cells began to differentiate assuming a fibro-chondrocytic phenotype. The fibrous matrix tended to be preserved both in the two horns and in the central body. After 14 days, some vessels were still visible in the inner zone in both treatments. In normoxia, cell death and hypertrophic phenomena were more pronounced. These findings differ from recent evidence, where the study of the entire tissue showed an onset of extracellular matrix production after 14 days of culture [38]. To explain these apparent conflicting data, we have to consider that the tissue cultured in the two experiments had different dimensions, and in the present study the smaller dimensions probably allowed for a better perfusion of the culture medium. There is no evidence in the literature to ascertain the cell viability of meniscus tissue-culture in hypoxia. In the present study, we observed that hypoxia allowed for maintaining a constant vitality index in the 14 days of culture in the two horns; in the central body it even seemed that hypoxia favored cell viability at the longest experimental time. Many studies have shown opposite findings, which demonstrated that hypoxia was able to inhibit cell proliferation of many cell types and lines [47,48,49,50,51,52,53,54,55,56,57,58]; however, more recent evidence in mini chromosome maintenance (MCM) proteins are investigating molecular mechanisms that allow for cell replication even in hypoxic conditions [59,60,61,62,63,64]. Hence, knots will be untied in the future with further experiments. To characterize the matrix, type I collagen, type II collagen, and SOX9 were evaluated. These analyses revealed that hypoxia accentuated the downregulation of COL1A1 in the three portions, but it acted particularly in the posterior horn, where the genes COL2A1 and SOX9 were upregulated after 14 days. These mechanisms were also reflected at the level of matrix proteins. These results represent an intermediate stage between previous studies, which were focused both on fresh animal tissue and on cell cultures. In fact, Di Giancamillo et al. characterized the protein composition of both newborn and adult pig meniscus [29]. They demonstrated that the tissue had a significant increase in COL2 and a significant decrease in SOX9 production in the transition from newborn to adult animals. Conversely, Adesida et al. made a comparison between adult human meniscus cells and articular chondrocytes in COL1A1, COL2A1, and SOX9 gene expression under hypoxic conditions [35]. In this case, the meniscus was analyzed by dividing the outer zone from the inner zone and they showed that the two areas have an opposite trend, especially as regards the expression of collagen genes. In the outer zone they demonstrated a downregulation of COL1A1 and an upregulation of COL2A1 and SOX9; whereas, in the inner zone they displayed an upregulation of COL1A1 and COL2A1. No significant changes were observed in SOX9 expression in the meniscus, and no significant changes were observed in chondrocytes. Moreover, in a previous study on the effect of hypoxia on meniscus, the same variations in the expression of collagens and SOX9 were observed. In this case, hypoxia was able to increase COL2A1 expression and reduce the expression of COL1A1 and SOX9 in a static tissue culture [38]. A new scenario for future meniscal studies involving hypoxia has been recently introduced by Lafont et al., who found that HIF-2α stabilization under hypoxia increases COL2A1 levels via SOX9 in healthy human cultured cartilage [65]. Moreover, the same authors found that, although SOX9 is crucial for tissue differentiation, hypoxia and more specifically HIF-2α, also promoted other SOX9-independent factors involved in cartilage homeostasis. These data on healthy cultured chondrocytes are suggestive of a better control of the cell phenotype and, hence, tissue repair: in the future it would be interesting to evaluate the possible role of HIF-2α stabilization on meniscus homeostasis.

In this work, particular attention was given to the expression of factors modulating the angiogenesis. We have seen that at the gene level, endostatin did not have any significant difference between normoxia and hypoxia; nevertheless, the immunohistochemical analyses suggested that the observed peaks of endostatin levels at T7 were sufficient to activate a cellular response, considering that the antibody labeling of the hypoxic tissue was particularly evident: this result may correspond to a cellular reaction to the hypoxic environment. Moreover, although the peaks in gene expression were slight, they matched to an opposite trend in the CD31 pro-angiogenic marker. We hypothesize that 14 days of tissue culture of the neonatal meniscus are not sufficient to trigger an adequately strong and diversified cellular response between the two treatments, in absence of biomechanical stimuli, which have not been applied in this study. However, in the first days of culture there was a visible increase in endostatin production, which could correspond to the cellular reaction to the hypoxic environment. The morphological changes reflect the data published by Pufe et al., where a positive immunostaining for endostatin was highlighted in human fetal cartilage and meniscus [21]. In our work, intense endostatin stain was associated with the absence of collagen type II signal in predominantly fibroblastic type cells. At this stage of development, the basal membrane of the vessels and the pericellular matrix of the meniscal cells were also positive for endostatin. On the other hand, in the adult human meniscus, Pufe et al. observed positivity in the pericellular matrix of fibrochondrocytes, but negativity in the extracellular matrix [21]. The same authors also studied fetal mouse meniscus, and they again showed an intense positivity for endostatin with a slight decrease after birth, and weak marking in the adult meniscus [55]. Feng et al. studied the function of endostatin in rabbit articular chondrocytes and they found that this factor can promote their proliferation [66]. These authors also demonstrated the homeostatic function of endostatin in cartilage, that works with a protective feedback mechanism, controlling local homeostasis during tissue remodeling. This hypothesis is also supported by Ergun et al., who suggested that endostatin reduced VEGF-induced formation of endothelial tubes and microvessels emerging from the aortic rings and it blocked their network [67].

To summarize, in this work we demonstrated that in in vitro culture, hypoxia retained the initial phenotype of the cells, delaying the degeneration associated with the static culture. Hypoxia acted preferentially on the posterior horn, increasing the production of extracellular matrix proteins (type II collagen and SOX9) towards a mature tissue phenotype. Furthermore, it favored the production of pro- and anti-angiogenic vascular factors which both tended to be upregulated in the early phase of culture. The results obtained suggest a positive role of hypoxia on the differentiation of meniscal tissue. These findings may thus open new frontiers in the field of meniscus tissue engineering, highlighting the crucial role of the vascular pathways in meniscal repair. Further studies involving biomechanical stimuli will be necessary to optimally mimic the normal physiology of the knee.

## 4. Materials and Methods

### 4.1. Preparation of Meniscal Tissue Cultures

**Figure 4:**
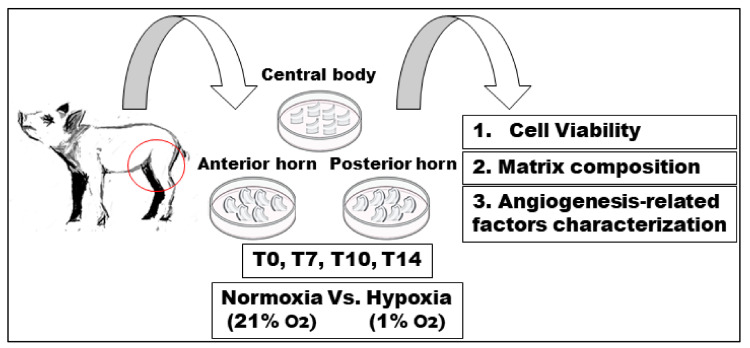
Experimental design. Medial menisci were harvested from neonatal pigs. Anterior, central, and posterior portions were separated and then cultured in normoxic or hypoxic conditions. Samples were collected at 0, 7, 10, 14 days, and analyzed for cell viability, matrix composition, and angiogenesis-related factors expression.

Forty-two knee joints of newborn pigs (Landrace × Large white), which had immediately died after farrowing under the weight of the mother or by natural causes, were obtained from a local farm and dissected to isolate the hind limbs. The project was approved by Ethic Committee University of Milan (OPBA, 58/2016). In the present study we chose the medial meniscus since 60–80% of the weight acts on the medial part of the knee joint [68]. The whole medial menisci were carefully isolated from both left and right knees in aseptic conditions. Menisci were harvested after the capsular tissue and ligaments were removed and each meniscus was transversally cut into three parts corresponding to the anterior horn (A), the central body (C), and the posterior horn (P). The samples were immediately placed in Dulbecco’s modified Eagle’s medium (DMEM, Thermo Fisher Scientific, Portland, OR, USA) with 10% fetal calf serum (FCS, Euroclone, Pavia, Italy), 1% glutamine (Thermo Fisher Scientific), 1% antibiotics (100 U/mL penicillin-streptomycin, Thermo Fisher Scientific); 50 μg/mL gentamicin sulfate, 0.5 μg/mL amphotericin B (Euroclone) for in vitro tissue culture experiments. The samples were exposed to normoxic (N) condition (at 37 °C in a 5% CO_2_/21% O_2_ humidified atmosphere) vs. hypoxic (H) condition (at 37 °C in a 5% CO_2_/1% O_2_ humidified atmosphere in a SCI-tive-workstation (Baker Ruskinn, Bridgen, UK). A, C, and P meniscal portions were collected at the following time points: day of meniscus explant from neonatal pig as day zero (T0, n = 6 menisci, each of which divided into A, C, and P, respectively), seven (T7, n = 12 menisci, each of which divided into A, C, and P, respectively), 10 (T10, n = 12 menisci, each of which divided into A, C, and P, respectively), and 14 (T14, n = 12 menisci, each of which divided into A, C, and P, respectively) days. Twenty-one menisci were analyzed by histology and immunohistochemistry and 21 menisci were investigated for quantitative real time PCR (Figure 4).

### 4.2. Morpho-Functional Analyses

#### 4.2.1. Histology

The samples were rinsed with phosphate buffer saline (PBS, Thermo Fisher Scientific) at the end of each time point to remove DMEM, then fixed in 10% (*v*/*v*) phosphate-buffered formaldehyde (Bio-Optica Milano, Milan, Italy) overnight at room temperature (RT). The samples were dehydrated in graded series of ethanol, cleared in xylene, and embedded in paraffin wax. Horizontal histological sections including both the inner and the outer meniscal portions were cut at 4 μm thickness.

Hematoxylin (Bio-Optica) and Eosin (Bio-Optica) staining was carried out for morphological evaluation on three representative sections (out of a total of 25) taken from the ventral, middle, and dorsal level of each meniscus, respectively. Images were acquired with a B-1000 optical microscope (OPTIKA, Bergamo, Italy).

#### 4.2.2. Quantitative Polymerase Chain Reaction (qPCR)

Menisci were homogenized with a tissue disperser (IKA, Cologne, Germany). Extraction of RNA was performed following instruction of RNeasy Mini Kit (Qiagen, Hilden, Germany). Quantity and quality of RNA were assessed using Nanodrop 8000 (ThermoFisher Scientific). RNA was reversely transcribed by using ImProm II reverse Transcription System (Promega, Milan, Italy). To assess cell viability, primers amplifying Proliferating Cell Nuclear Antigen (PCNA) and Caspase 3 (CASP3) were used (Table 1). cDNA was amplified with PowerUp SYBR master mix (ThermoFisher Scientific) on 7500 Fast Realtime PCR System (Applied Biosystems, Foster City, CA, USA). The primers were designed by Primer-BLAST tool (https://www.ncbi.nlm.nih.gov/tools/primer-blast/ last access: 29 April 2021) and supplied by Eurofins Genomics (Eurofins Genomics, Ebersberg, Germany). The reactions were performed in three stages: (1) holding stage initializing at 50 °C for 20 s, then 95 °C for 10 min; (2) cycling stage at 95 °C for 15 s, then 60 °C for 1 min (the cycling stage was repeated for 40 cycles); (3) melting curve stage at 95 °C for 15 s, followed by 60 °C for 1 min, 95 °C for 30 s, and 60 °C for 15 s.

The mRNA expression values were calculated as relative quantities normalized to β-actin. Data were analyzed according to a comparative method [69], as fold change (2−ΔΔCt value) with ∆Ct = [Ct (gene of interest) − Ct (β-actin)] and ∆∆Ct = [(∆Ct at day n) − (∆Ct at day 0)], where n = number of days of culture.

### 4.3. Matrix Characterization

#### 4.3.1. Histology

To distinguish collagen type I fibers, Picrosirius Red staining was performed. Picrosirius Red marks collagen type I in yellow-orange color, in contrast with red staining for collagen non-type I content. Briefly, sections were immersed in Picrosirius Red staining solution (Abcam Laboratories, Cambridge, UK) for 60 min, then rapidly rinsed twice in 5% acetic acid solution, and dehydrated in absolute ethanol. Sections were dehydrated end cleared, and finally sealed on coverslips using Eukitt mounting medium (Sigma-Aldrich, St. Louis, MA, USA). Images were acquired with polarized C-P3 Optika Microscope (OPTIKA, Bergamo, Italy).

#### 4.3.2. Immunohistochemistry

De-waxed sections were submerged in 3% (*v*/*v*) H_2_O_2_ for 30 min to block the endogenous peroxidase. Heat-induced antigen retrieval in citrate buffer pH 6 or Tris/EDTA buffer pH 9 for 20 min was carried out when required, then sections were incubated with hyaluronidase type II (2 mg/mL, Sigma-Aldrich) in PBS (Thermo Fisher Scientific) pH 6 for 30 min at 37 °C. Blocking of non-specific epitope binding was performed by incubating the sections in 10% (*v*/*v*) normal goat serum (NGS, Thermo Fisher Scientific) in PBS for 1 h at RT. The sections were incubated with primary antibodies (anti-COL2 and anti-SOX9, Abcam) diluted in 5% (*v*/*v*) NGS in PBS overnight at 4 °C in a humid chamber, then rinsed three times in PBS for 10 min, and finally incubated with goat anti-rabbit IgG biotinylated (Vector Laboratories Inc., Burlingame, CA, USA) antibodies diluted 1:100 in 5% (*v*/*v*) NGS in PBS for 1 h at RT. The sections were washed in PBS 5 min three times, subsequently incubated in Vectastain Elite ABC-HRP kit Peroxidase (Vector Laboratories Inc.) for 1 h at RT. Finally, after rinsing in PBS 5 min for three times, 3,3′-diaminobenzidine (DAB) (Thermo Fisher Scientific) was used as a chromogen reporter. The sections were counterstained with Harris hematoxylin (Bio-Optica) for ten seconds, subsequently dehydrated end cleared, and ultimately sealed on coverslips using Eukitt mounting medium (Sigma-Aldrich). The specificity of immunohistochemistry reaction for all antibodies was proved either by using a negative control (i.e., the omission of the specific primary antibodies resulting as negative staining), and a positive control (i.e., other tissues that are known to contain the target molecule). Images were acquired with a B-1000 Optika microscope (OPTIKA).

### 4.4. qPCR

To evaluate gene expression of extracellular matrix proteins such as collagen type I and type II, primers for COL1A1, COL2A1, and SOX9 (Table 1) were used for qPCR (material and methods are described above in the matrix characterization paragraph).

### 4.5. Vascular-Related Markers Characterization

#### Immunohistochemistry

Polyclonal anti-endostatin (ENDO, Millipore, Temecula, CA, USA) and polyclonal anti-CD31 (Abcam) antibodies were used. Anti-ENDO antibody marked anti-angiogenic protein, while anti-CD31 antibodies identified pro-angiogenic protein (immunohistochemical protocol is described previously in the matrix characterization paragraph).

### 4.6. qPCR

To evaluate anti-angiogenic and pro-angiogenic vascular gene expression, endostatin (COL18A1) and platelet and endothelial cell adhesion molecule 1 (CD31) primers (Table 1) were used for qPCR (material and methods are described previously in the matrix characterization paragraph).

### 4.7. Statistical Analysis

Statistical analysis was performed with SAS statistical software (ver. 9.3, Cary, NC, USA). Data from qRT-PCR analyses were analyzed using 2-way ANOVA with time (0, 7, 10, and 14 days) and treatment (normoxia or hypoxia) as main factors. The individual medial meniscus of each piglet was considered as the experimental unit. The data are presented as means with standard errors. Differences between means were considered significant at *p* < 0.05, *p* < 0.01, and *p* < 0.001.

## 5. Conclusions

Our study demonstrated that in this in vitro culture, hypoxia retained the initial phenotype of the cells, delaying the degeneration associated with the static culture. Hypoxia acted preferentially on the posterior horn, increasing the production of extracellular matrix proteins (type II collagen and SOX9) towards a mature tissue phenotype. Furthermore, it favored the production of pro- and anti-angiogenic vascular factors, which both tended to be upregulated in the early phase of this culture.

## Figures and Tables

**Table 1:** Primer sequences.

Gene	Forward Sequence (5′-3′)	Reverse Sequence (5′-3′)	Amplicon Size (bp)
Actin Beta (*ACTB*)	CAA GGA GAA GCT CTG CTA CG	AGA GGT CCT TCC TGA TGT CC	245
Caspase 3 (*CASP3*)	TGG GAT TGA GAC GGA CAG TG	CGC TGC ACA AAG TGA CTG GA	121
Collagen Type I Alpha 1 Chain (*COL1A1*)	CCA ACA AGG CCA AGA AGA AG	ATG GTA CCT GAG GCC GTT CT	64
Collagen Type II Alpha 1 Chain (*COL2A1*)	CAC GGA TGG TCC CAA AGG	ATA CCA GCA GCT CCC CTC T	102
Endostatin (*COL18A1*)	GCC GAC TTC CAG TGC TTC	CGT CGC ACG ATG CTG TAG	104
Platelet And Endothelial Cell Adhesion Molecule 1 (*CD31*)	CAC CGA GGT CTG GGA	CGG AGC CTT CCG TTC	103
Proliferating Cell Nuclear Antigen (*PCNA*)	GCA GAG CAT GGA CTC GTC TC	TTG GAC ATG CTG GTG AGG TT	120
SRY-Box Transcription Factor 9 (*SOX9*)	CCG GTG CGC GTC AAC	TGC AGG TGC GGG TAC TGAT	119

## Data Availability

Publicly available datasets were analyzed in this study. This data can be found here: https://osf.io/yvnaw/?view_only=0f3f0ac9f362474eb52a4845125558f5. Last access: 7 October 2021.
